# Multiple Network Disconnection in Anosognosia for Hemiplegia

**DOI:** 10.3389/fnsys.2020.00021

**Published:** 2020-04-29

**Authors:** Elena Monai, Francesca Bernocchi, Marta Bisio, Antonio Luigi Bisogno, Alessandro Salvalaggio, Maurizio Corbetta

**Affiliations:** ^1^Department of Neuroscience, Neurological Clinic, University of Padua, Padua, Italy; ^2^Padova Neuroscience Center, University of Padua, Padua, Italy; ^3^Department of Neurology, Radiology, and Neuroscience, Washington University in St. Louis, St. Louis, MO, United States

**Keywords:** anosognosia, hemiplegia, stroke, structural disconnection, network, awareness

## Abstract

Anosognosia for hemiplegia (AHP) is a complex syndrome whose neural correlates are still under investigation. One hypothesis, mainly based on lesion mapping studies, is that AHP reflects a breakdown of neural systems of the right hemisphere involved in motor function. However, more recent theories have suggested that AHP may represent a disorder of cognitive systems involved in belief updating, self-referential or body processing. Two recent studies, using a method to estimate the degree of white matter disconnection from lesions, have indeed shown that patients with AHP suffer from damage of several long-range white matter pathways in association cortex. Here, we use a similar indirect disconnection approach to study a group of patients with motor deficits without anosognosia (hemiparesis or hemiplegia, HP, *n* = 35), or motor deficits with AHP (*n* = 28). The HP lesions came from a database of stroke patients, while cases of AHP were selected from the published literature. Lesions were traced into an atlas from illustrations of the publications using a standard method. There was no region in the brain that was more damaged in AHP than HP. In terms of structural connectivity, AHP patients had a similar pattern of disconnection of motor pathways to HP patients. However, AHP patients also showed significant disconnection of the right temporo-parietal junction, right insula, right lateral and medial prefrontal cortex. These associative cortical regions were connected through several white matter tracts, including superior longitudinal fasciculus III, arcuate, fronto-insular, frontal inferior longitudinal, and frontal aslant. These tracts connected regions of different cognitive networks: default, ventral attention, and cingulo-opercular. These results were not controlled for clinical variables as concomitant symptoms and other disorders of body representation were not always available for co-variate analysis. In conclusion, we confirm recent studies of disconnection demonstrating that AHP is not limited to dysfunction of motor systems, but involves a much wider set of large-scale cortical networks.

## Introduction

*Anosognosia*, or lack of awareness of having a disorder or disability (Mograbi and Morris, 2018) represents an impressive phenomenon whose neural correlates have not been completely clarified.

*Anosognosia for hemiplegia (AHP)* refers to a syndrome in which a patient, typically following a stroke, fails to recognize his motor deficit. Patients may explain away the motor deficits with other reasons (a fall, arthritis, unwillingness to move at this time, etc.). AHP after stroke is the most investigated form of anosognosia, and it is clinically relevant due to its negative impact on motor rehabilitation (Vocat et al., 2010). Other forms of anosognosia have been described: for visual stimuli (hemianopia and Anton’s syndrome), spatial and body processing, and cognitive deficits (Goldenberg et al., 1995; Anton, 1898; Barrett et al., 2005; Spinazzola et al., 2008; Vossel et al., 2012; Ronchi et al., 2014; Baier et al., 2015).

Traditionally, anosognosia is considered an alteration of monitoring systems specific for the involved function. For instance, monitoring systems for movement in the case of AHP. This view is supported by clinical reports showing dissociation of awareness between different types of deficits in the same patient. For instance, a patient may ignore one type of deficit, but be aware of other types of deficits. Also anosognosia can be separated from general cognitive impairment (Bisiach et al., 1986; Berti et al., 1996; Spinazzola et al., 2008).

Motor control and movement awareness of intended motor acts have been proposed to reside within a frontal-parietal circuit (Haggard, 2005), involving mainly premotor cortex (PMC), supplementary motor area (SMA), posterior parietal cortex, and prefrontal cortex (PFC; Frith et al., 2000; Desmurget and Sirigu, 2009). These regions code intended, predicted, and actual movement states through feedforward and feedback signals.

Anatomo-clinical studies of AHP have reported damage of several fronto-temporo-parietal regions, as well as insula and subcortical regions. As an example, some studies have emphasized the importance of impaired sensory feedback with spared motor intentions or lack of a feedforward motor plan or an impairment of a motor “*comparator*” between an internal prediction and actual action (Heilman, 1991; Berti et al., 2005; Fotopoulou et al., 2008; Spinazzola et al., 2008; Heilman, 2014) involving premotor, sensory-motor regions, temporo-parietal junction (TPJ), basal ganglia, PFC, and insular cortex.

However, more recently, other theories have suggested that, at least for AHP, other systems beyond the motor system must be involved: e.g., systems involved in perspective taking (Besharati et al., 2016), reality checking and belief updating (Vuilleumier, 2004; Jenkinson et al., 2010; Vocat et al., 2013), and top-down and bottom-up processes on the prediction of the current state of the body (Fotopoulou, 2014). For instance, damage of the insula in AHP has been interpreted as a body representation and body schema disorder (Craig, 2002; Karnath, 2005; Craig, 2009). Beyond sensory-motor systems Vocat et al. (2013) have suggested a deficit of cognitive control mechanisms through damage of PFC and fronto-striatal circuits.

Recent advances in network neuroscience have emphasized the critical role of distributed interacting cognitive systems. Similarly, cognitive deficits in stroke have been associated with widespread network dysfunction, and white matter pathways damage Kang et al. (2003); Wessels et al. (2006); Boes et al. (2015); Corbetta et al. (2015); Ramsey et al. (2017). Similarly, for AHP a network view has been proposed (Fotopoulou, 2014) in which multiple cognitive system contribute in addition to the sensory-motor system to the syndrome.

Two recent studies of AHP are particularly relevant to our own work. Moro et al. (2016) identified using a voxel-based lesion analysis damage to several white matter regions near/at corona radiata, arcuate fasciculus and ventral superior longitudinal fasciculus, along with cortical damage of the rolandic operculum (ventral premotor cortex), insula, Heschl and superior temporal giry, and subcortically, basal ganglia. Persistent anosognosia chronically was associated with damage of fronto-temporal cortex (ventral premotor cortex, Heschl and temporal superior cortex), as well as long white matter tracts (cortico-spinal tract, anterior segment of fasciculus arcuate, and corpus callosum).

Pacella et al. (2019) used a structural disconnection approach (described below) to study network correlates of AHP. They found disconnection of white matter pathways that connected three sets of networks: a premotor loop, the limbic system, and the ventral attention network (VAN). In terms of pathways, AHP lesions disconnected primarily cingulate bundle (limbic), SLF III (VAN), and tracts connecting with pre-SMA such as frontal aslant and fronto-striatal (premotor loop).

Our study along the same lines aims to study the neural correlates of AHP both in terms of lesion topography and white matter disconnection. First, we searched the literature for reports on AHP in which the picture of the lesions were shown (see section “Materials and Methods” for search criteria). Next, we traced each lesion in an atlas space (MNI-152) using the approach of Boes et al. (2015) validated for functional connectivity in several recent publications (Laganiere et al., 2016; Darby et al., 2017, 2018; Cohen et al., 2019). Neuropsychological information on these cases was incomplete, and could not be used for analysis. Third, as in Pacella et al. (2019), we used an indirect estimate of white matter disconnection obtained by embedding a patient’s lesion into an atlas of white matter connections derived from healthy subjects. This approach, developed by Foulon et al. (2018), allows not only to estimate group-wide differences in structural disconnection, but also to identify the cortical regions corresponding to the disconnected white matter pathways. To identify AHP specific lesions or disconnection correlates we compared AHP patients with a group of stroke controls with significant motor impairment (hemiparesis or hemiplegia) from Corbetta et al. (2015) for whom the lesion volumes were available.

## Materials and Methods

### Subjects

The electronic database MEDLINE (accessed through PubMed) was searched. The search was conducted in April 2018 and consisted of all the following terms related to several body-self disorders:/*anosognosia, stroke*//*anosognosia, hemiplegia, stroke*//*asomatognosia stroke*//*misoplegia stroke*/,/*out of body experience stroke*//*personal neglect stroke*//*somatoparaphrenia stroke*/. Seven hundred and nineteen articles were examined from which we included patients with anosognosia for left hemiplegia after right-hemisphere stroke and with available pictures of lesions in the report.

Twenty-eight subjects from 21 papers were included. Two subjects were not selected because of the poor quality of the published images. Among the selected articles, 20 described single cases, providing demographical and neurological scores for each patient, whereas one article included a group analysis with mean demographical and neurological scores, but included lesion imaging for each patient (Cappa et al., 1987; House and Hodges, 1988; Berti and Frassinetti, 2000; Tei, 2000; Morin et al., 2003; Venneri and Shanks, 2004; Turnbull et al., 2005; Fotopoulou et al., 2008, 2009, 2011; van Stralen et al., 2011; Cogliano et al., 2012; Venneri et al., 2012; Besharati et al., 2015; Di Vita et al., 2015; Moro et al., 2015; Piedimonte et al., 2015, 2016; Salvato et al., 2016; Facchin and Beschin, 2018).

Among the 28 selected subjects (mean age 69,85 ± 12 years, range 51–93; 11 women, 11 men and 6 not specified, 14 lesions were documented with original CT scans, 6 lesions with original MRI, 6 with reconstruction on an MNI template, two of them in Damasio atlas spaces. All 28 subjects had single lesions, of which 12 ischemic, 7 hemorrhagic, while for 9 subjects the etiology was not specified.

A control group of first time stroke subjects with motor impairment, hemiparesis or hemiplegia (HP), was selected from the cohort (*n* = 132) described in Corbetta et al. (2015). We selected subjects with an overall motor impairment greater than one standard deviation from control subjects based on a principal component analysis of motor scores across different tasks of strength, coordination and dexterity (see Corbetta et al., 2015 for how the overall motor score was calculated). There were 19 subjects with right hemisphere lesions and 16 subjects with left hemisphere lesions. Left hemisphere lesions were flipped to the right hemisphere for comparison with the AHP group that was comprised all of right hemisphere lesions. The control (HP) group in the end included *n* = 35 subjects (mean age 55,6 ± 9,0 years, range 38–83, 18 women, 17 men, Table 1). All 35 control subjects underwent an MRI protocol as specified in Corbetta et al. (2015).

**TABLE 1:** Demographical, clinical and radiological data of AHP and HP patients.

Demographic, clinical and radiological data	AHP patients	HP patients
Selected subjects	28	35
Side of lesion R/L	28/0	19/16
Mean age (Year)	69.85 ± 12	55,6 ± 9,0
Sex F/M	11/11, 6 NS	17/18
CT scan	14	NS
MRI	6	35
Reconstruction (MNI/Damasio)	8	0
Mean imaging- delay after stroke (D)	91,0 ± 191,7	14,00 ± 5,57
Lesion Volume (mm^3^)	26610,8 ± 28693,1	74334,4 ± 84587,2
Type of stroke I/H	12/7, 9 NS	25/8, 2 I + H

*NS, not specified; I, ischemic; H, haemorrhagic; I + H, ischemic with haemorrhagic infarction.*

Scanning was performed with a Siemens 3T Tim-Trio scanner at the School of Medicine of the Washington University in St. Louis, and included sagittal MP-RAGE T1-weighted image. Lesion segmentation was described in Corbetta et al. (2015). Individual T1 MRI images were registered to the Montreal Neurological Institute brain using FSL (FMRIB Software Library) FNIRT (FMRIB non-linear imaging registration tool^1^). Lesions were manually segmented using the Analyze biomedical imaging software system^2^ (Robb and Hanson, 1991).

More comprehensive demographical, clinical and radiological data of AHP patients and HP controls are reported in Tables 1, 2 and Supplementary Table 1.

**TABLE 2:** Clinical features of AHP and HP patients.

Clinical feature	AHP patients	HP patients
Selected subjects	28	35
Sensory impairment	14	20
Visual impairment	12	8
Neglect	22	12
Somatoparaphrenia	7	0
Asomatognosia	7	0
Anton syndrome/anosognosia for visual impairment	2	0
Anosognosia for hemianesthesia	3	0
Others*	6	0

*e.g., anosognosia for neglect, allochiria, anosodiaphoria, hyperkinetic motor behavior, halien hand, and misoplegia. 0 if absent or not specified.

### Drawing of the Lesions

Brain lesions were drawn onto a standard brain template from FSL^3^ (MNI152 brain, 1 mm × 1mm × 1 mm) using itk-SNAP as lesion mapping software.^4^ Lesions from published papers were traced manually in the 2D axial plane of the template brain using neuroanatomical landmarks for guidance. The template was re-oriented to fit the plan of the published picture to increase accuracy. The lesions were segmented by medical students (FB, ALB) and neurology residents (EM, AS) and were all checked by a board-certified neurologist (MC). In case of lesion with multiple slices they were traced in the same atlas space (see Supplementary Material). The drawn lesions were subsequently down-sampled to a 2 mm × 2 mm × 2mm 3D space^5^ (MNI152 brain, 2 mm × 2 mm × 2 mm). Lesions of the control group from Corbetta et al. (2015) were already available having been previously segmented and normalized to the MNI152 brain template^6^ (MNI152 brain, 2 mm × 2 mm × 2 mm). Lesion volume was calculated for each subject in the AHP and HP groups.

### Lesion-Based Analyses

The distribution of the lesions was assessed at the voxel-level in the two groups^7^ (FSL Version 5.0.11). A two-sample unpaired *T*-test with lesion size as a co-variate was performed between AHP and HP groups applying the general linear model (GLM) and randomize function (with 5000 permutations; Winkler et al., 2014), tools from the FMRIB Software Library^8^ (FSL Version 5.0.11) package (Smith et al., 2004). A threshold-free cluster enhancement approach was applied to control for family-wise error.

### Structural Disconnection Analyses (Disconnectome)

Disconnectome maps were calculated using BCBtoolkit (Foulon et al., 2018). In this approach a dataset of 164 diffusion weighted imaging data set from healthy controls from the Human Connectome Project (Rojkova et al., 2016) was used to track fibers passing through each lesion. For each participant tractography was estimated as described in De Schotten et al. (2011). The lesions in MNI152 space were registered to each control native space using affine and diffeomorphic deformations (Klein et al., 2009; Avants et al., 2011) and used as seed for the tractography in Trackvis (Wang et al., 2007). Tractography from the lesions were transformed in visitation maps (De Schotten et al., 2011), binarized, and brought to the MNI152 using the inverse of precedent deformations. Finally, a percentage overlap map was produced by summing at each point in MNI space the normalized visitation map of each healthy subject. Hence, in the resulting disconnectome map, the value of each voxel takes into account the inter-individual variability of tract reconstructions in controls and indicate a probability of disconnection from 0 to 1 for a given lesion (Thiebaut de Schotten et al., 2015). Then a threshold of 0.5 was applied, considering a tract involved when the probability to be present in a given voxel was estimated above 50% (Thiebaut De Schotten et al., 2014).

This procedure was replicated for all AHP and HP lesions, allowing the construction of a disconnectome map for each patient in both groups. These steps were automatized in the tool/disconnectome/as part of the BCBtoolkit. A two-sample unpaired *T*-test with lesion size as covariate between groups was carried out controlling for family-wise error using a threshold-free cluster enhancement approach.

This analysis allows to obtain a map (*disconnection-based map*) of the statistically significant group differences in structural disconnection.

A concomitant analysis with the same procedure was also conducted comparing AHP patients with *n* = 19 right hemisphere only HP patients (see Supplementary Materials).

To examine the overlap of the disconnection maps with the normal white matter tracts, cortical parcels, or subcortical parcels, two measures of overlap were computed as follows:

$$\%\mathrm{disconnected}\mathrm{tract}=\frac{N_{d}\,\cap N_{t}}{N_{t}}\times 100$$

$$\%\mathrm{disco}/\mathrm{parcel}\mathrm{overlap}=\frac{N_{d}\,\cap N_{t}}{N_{d}}\times 100$$

where *N*_*d*_ is the number of voxels belonging to the whole *disconnection-based map* and *N*_*t*_ the number of voxels belonging to each specific white matter tract/brain region/parcel, specified by *t*.

% Disco/parcel overlap and% disconnected tract refer, respectively, to the overlap of cortical parcels computed in relation to the total number of voxels composing the *disconnection-based map* and the total number of voxels involved by the disconnection belonging to specific white matter tract.

The variable *t* defines regions of interest in three atlases, specifically: (i) a white matter tracts atlas (Rojkova et al., 2016); (ii) a subcortical atlas composed of thalamus, caudate, putamen, pallidum, brain-stem, hippocampus, amygdala and accumbens (part of FSL); and (iii) the Gordon–Laumann parcellation for the cortical surface (Gordon et al., 2016). All Gordon–Laumann networks were analyzed, only those that contribute above 2% to the whole disconnection were selected.

## Results

### Lesions and Disconnection Maps in AHP and HP Group

The distribution of lesions was similar in the two groups, involving frontal, temporal, and parietal lobes, as well as insular cortex and subcortical regions in the basal ganglia and thalamus. The brainstem was involved only in the HP group.

**FIGURE 1:**
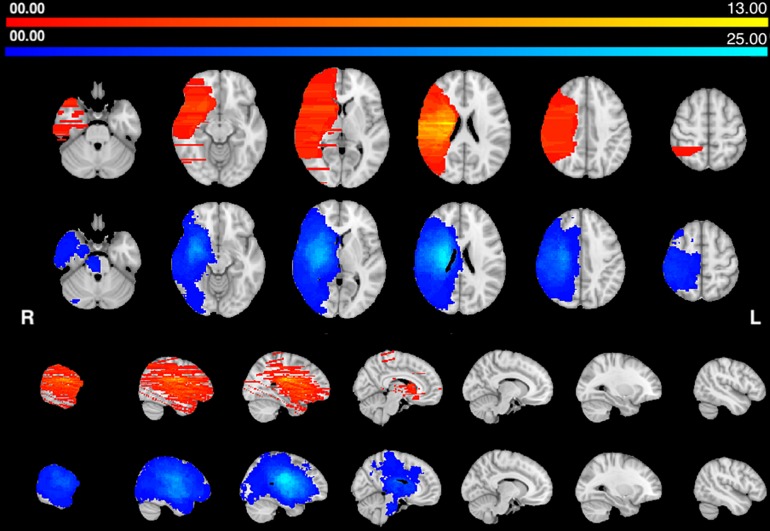
Lesions frequency map: Voxel lesion overlap in AHP (orange-yellow) and HP groups (blue-teal). The color scale indicates the max number of patients with lesions in one voxel.

The center of damage in the HP group was in the basal ganglia and central white matter similarly to Corbetta et al. (2015). The center of damage in the AHP group was more diffuse with the most common locations of damage in the frontal and parietal white matter (Figure 1). Note that the distribution of lesions is uneven and discontinuous across the brain volume in the AHP group due to the uneven sampling of the published pictures (as described in section “Materials and Methods”).

**FIGURE 2:**
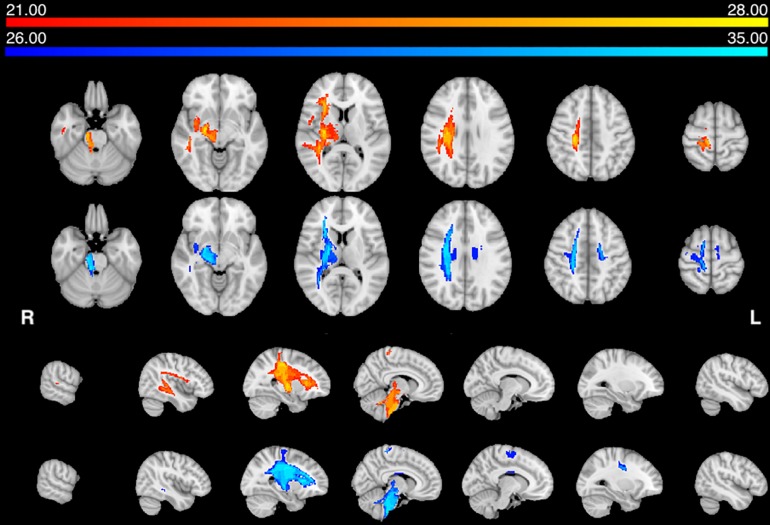
Structural disconnection frequency map (75%): Voxels of white matter tract overlap in AHP (orange–yellow) and HP (blue-teal) groups. The color scale indicates max number of patients per voxel.

We computed the structural connectivity disconnection map that was common for each group, by thresholding the group maps at 75% overlap, i.e., 75% of the patients showed the same disconnection at that location. Both groups of patients showed a similar disconnection of the dorsal white matter pathways descending from the motor/premotor/parietal cortex to the internal capsule, and cerebral peduncle. There is also involvement of frontal and temporal white matter tracts, thalamo-cortical and basal ganglia-cortical tracts (Figure 2).

In summary, both groups have lesions located in the middle cerebral artery distribution, deep and superficial. They also show a common disconnection of descending motor pathways. In the HP group there is also a transcallosal (pre)-motor contralateral disconnection.

### AHP vs. HP Lesion vs. Disconnection Maps

**FIGURE 3:**
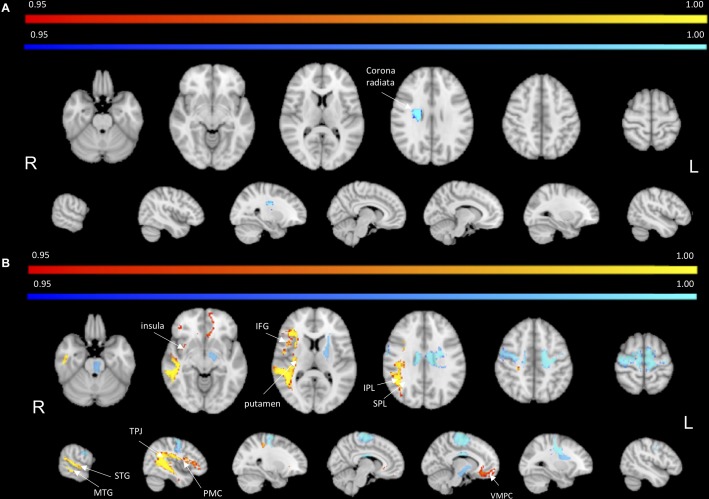
Lesion vs. disconnection maps. **(A)** Lesion-based mapping: voxels with significant difference in lesion frequency between HP and AHP patients. HP > AHP (*p* < 0.05) (blue-teal). No voxels for AHP > HP damage. **(B)** Disconnection-based maps: voxels showing significant difference in white matter disconnection. HP > AHP (*p* < 0.05) (blue-teal). AHP > HP (*p* < 0.05) (orange–yellow).

Next, we statistically compared the two groups in terms of lesion location and volume (*p* < 0.05 after correction for family-wise error). We did not find any significant difference in favor of the AHP group; however, there was significantly more damage in the dorsal corona radiata underlying the PMC in the HP group (Figure 3A).

In contrast, the structural disconnection analysis showed significant group differences (*p* < 0.05 after correction for family-wise errors). HP patients showed greater disconnection of the contralateral dorsal white matter underlying premotor and motor cortex and cortico-spinal tracts (Figure 3B). The AHP patients showed greater disconnection that involved the white matter underlying the superior and inferior parietal lobule (SPL, IPL), superior and middle temporal gyrus (STG, MTG), PMC, insula, and anteriorly in the inferior frontal gyrus (IFG). There was also a slight bilateral disconnection of medial and orbito-frontal cortex through the anterior segment of the corpus callous (genu; Figure 3B).

### Mapping Structural Disconnection Maps Onto White Matter Tracts, Subcortical Regions, and Cortical Networks

**FIGURE 4:**
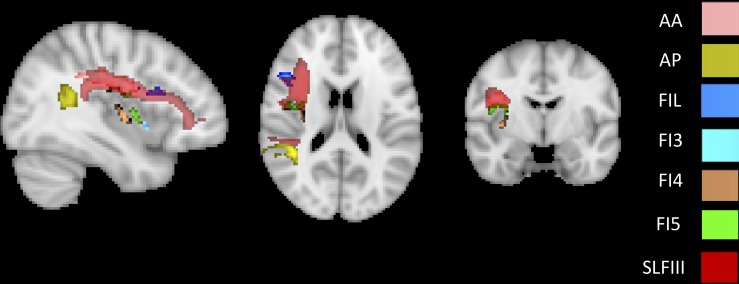
White matter tracts more commonly disconnected in AHP: main white matter tracts involved in AHP disconnection map as compared to HP. FI: fronto-insular tract; SLF III: superior longitudinal fasciculus III; AA and AP: anterior and posterior segment of arcute fasciculus; FIL: frontal inferior longitudinal fasciculus.

**FIGURE 5:**
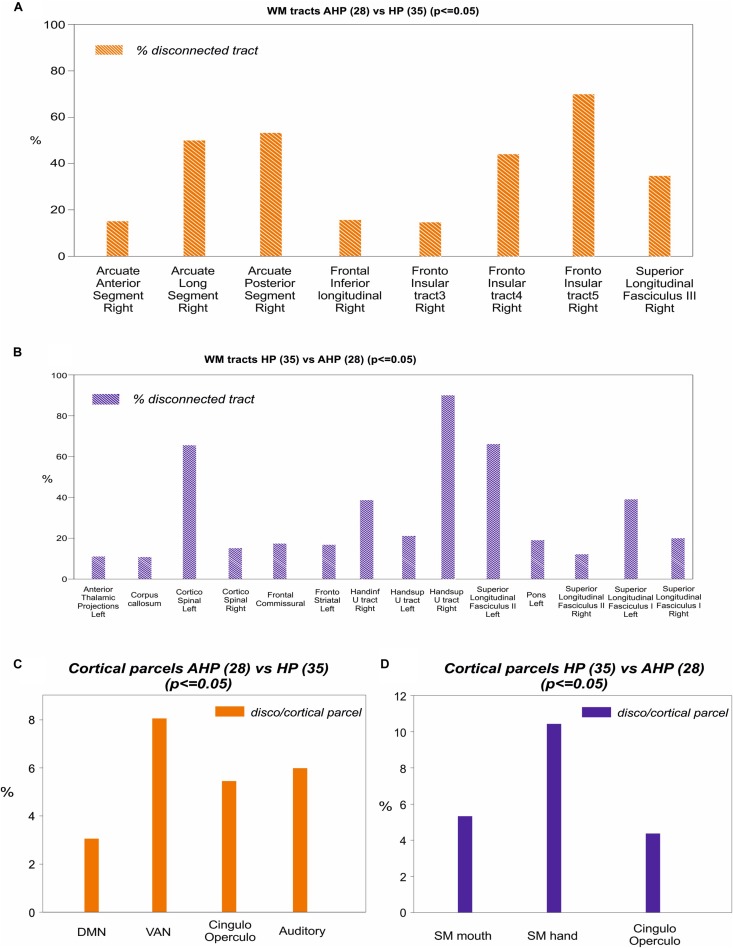
Mapping of disconnection maps on white matter tracts and cortical networks. **(A)** AHP vs HP: individual white matter tracts’ overlap with disconnectome map (>10% disconnected tract, orange bar). **(B)** Same for HP vs AHP (in blue). **(C)** AHP vs HP: % overlap > 2% with cortical parcellation networks based on the Gordon–Laumann atlas (disco/cortical parcel, orange bar). **(D)** Same for HP vs AHP (in blue).

To identify the white matter tracts involved in the map of structural disconnection, we computed the overlap between the disconnection maps and an atlas of the white matter tracts. The tracts more affected in AHP included the superior longitudinal III and frontal inferior longitudinal fascicles (SLF III, FIL), the arcuate fascicle (AF, mainly the posterior segment, AP but also the anterior segment, AA), the fronto-insular tracts (FI), and fronto-aslant tract (FAT; Figures 4, 5A).

In contrast, in the HP group, the white matter tracts more affected localized almost to motor pathways (cortico-spinal, pons), transcallosal connections and superior longitudinal fasciculus I and II(SLF I-II; Figure 5B).

Finally, we analyzed the cortical projections of the affected tracts in terms of brain networks. Regions disconnected in the AHP group belonged mainly to the VAN, auditory, cingulo-opercular (CON) and default mode network (DMN; Figure 5C).

## Discussion

### Methodological Issues and Limits of the Study

This study has many limitations. The first main limitation is that we drew manually the lesions in a template atlas from published pictures in the literature of different AHP cases and groups of patients. This approach was originally proposed by Boes et al. (2015) to investigate functional connectivity disconnection, what they call lesion symptom mapping (Fox, 2018), in rare syndromes or in patients in which direct measures of structural-functional connectivity are not available. We used these manually drawn lesions for a structural disconnection analysis, as proposed by Foulon et al. (2018). As far as we know, this is the first time these two methods have been combined, even though each method has already been separately applied.

In our hands, we first identified the best orientation of the atlas that would fit the published figure/s. This works best when multiple slices are displayed. Considerable uncertainty remains when only one slice is available. The pictures, first drawn by the medical students involved in the study (FB, ALB), were then checked by the neurology residents (EM, AS), and finally by a fully certified neurologist (MC). We did not perform formal test–retest analyses.

Usually published images of lesions include only one or few representative slides that do not capture the extent of the whole lesion. Therefore, lesion volume is on average underestimated. As a result, average lesion maps in the AHP group were discontinuous with gaps especially in the *z*-axis (Figure 1). This makes a statistical analysis at the group level problematic since the two groups do not uniformly cover the same brain volume. Moreover, on average, lesions in the HP group were larger in volume (Table 1). This difference is likely due to the differences in the way we identified the lesions, while in fact the opposite may be true. In fact lesions producing AHP (and hemiplegia) shall be larger, but also more variable in location than lesions producing HP only. The fact that AHP lesions, as drawn, are smaller and more variable shall favor the HP group in terms of lesion topography and disconnection. In fact, we found, for the HP group, more damage in the corona radiata, likely because of the more focal damage, and greater disconnection of motor/premotor/callosal connectivity in the central motor region. However, the disconnection for association pathways was greater in the AHP group suggesting a real biological effect.

Another limitation is the scarcity of clinical information in the AHP group. The published reports we used for this investigation often did not contain detailed information on non-motor deficits, such as neglect symptoms. In addition, AHP is typically measured using clinical scales (e.g., Bisiach et al., 1986) that often were not even available in all reports. Therefore, it was not possible to perform any correlation with neuropsychological scores, also to covary out possible contribution from other deficits.

Some of AHP patients showed other forms of alteration of bodily awareness, e.g., asomatognosia, i.e., a loss of recognition or awareness of part of the body; somato-paraphrenia, i.e., the hallucinatory experience of attributing one own’s arm to someone else or the subjective feeling of distortion or additional arms, and other forms of anosognosia (Table 2). So, a possible influence of the neural correlates of different syndromes and concomitant deficits with possible overlap or distinction could not be excluded from the performed analysis and represents a relevant limit of the present study.

Moreover, in the control group, anosognosia was not explicitly measured, even though it was reported by the expert clinical examiner of the trial. Most patients in the control group were examined at 2 weeks, a time in which AHP is disappearing.

Also, we cannot guarantee that the degree of motor deficit in AHP was the same as in the control group. For HP controls, we used a cut-off of *z* < -1 that included both mild, moderate, and severe patients (mean *z* score -2.10, SD 0.46).

The timing of brain scans was not uniform between the two groups. It was consistently at two weeks in the HP group, but was more variable in the AHP group (see Supplementary Table 1).

Finally, to increase the size of the control group we flipped the left side lesions onto the right hemisphere, hence abolishing potential hemispheric difference. However, a control analysis that compared directly AHP patients with *n* = 19 right hemisphere only HP patients yielded similar results (see Supplementary Figures 2–5 and Supplementary Tables 2, 3).

We performed a group-comparison analysis, not a single subject analysis, after thresholding to 0.9, that is 90% of healthy subjects showed that tract. The disconnection of different tracts was computed both as% of the whole disconnection, an index which favors white matter tract that have larger volume, as well as% disconnection of different tract, an index that takes into account also small tracts. Issues of thresholds are relevant and is hard to account for the relative behavioral effect of complete vs. incomplete disconnection.

### A Network Perspective on AHP

Previous anatomical studies related AHP to lateral premotor and prefrontal, TPJ, insula, and basal ganglia lesions (Bisiach et al., 1986; Starkstein et al., 1992; Ellis and Small, 1997; Pia et al., 2004; Berti et al., 2005; Karnath, 2005; Vocat et al., 2010).

For instance a review by Pia et al. (2004, p. 367), who reviewed a large number of studies concluded: “It seems to be equally frequent when the damage is confined to frontal, parietal or temporal cortical structures, and may also emerge as a consequence of subcortical lesions. Interestingly, the probability of occurrence of anosognosia is highest when the lesion involves parietal *and* frontal structures in combination, if compared to other combinations of lesioned areas. This pattern of lesions suggests the existence of a complex cortico-subcortical circuit underlying awareness of motor acts that, if damaged, can give raise to the anosognosic symptoms.”

Our distribution of lesions was compatible with the literature, and the center of damage was in the basal ganglia, insular and inferior frontal cortex and underlying fronto-temporal white matter tracts (Figure 1). However, the statistical analysis did not find any region that was differentially more affected in AHP than HP. In contrast, we found more frequent damage of the right dorsal corona radiata underlying motor and premotor regions in HP patients (Figure 3A). As noted above, this finding may be related to differences in the method used for lesion segmentation in the two groups. Interestingly, in the motor system, we also found some differences with greater disconnection of the corona radiata, and motor/premotor regions bilaterally in the HP group, consistent with the lesion results.

The most interesting result of this study was the difference in structural disconnection vis-a-vis a relative lack of difference in lesion location in favor of AHP. AHP patients showed a much more widespread pattern of white matter disconnection in the ipsilateral hemisphere reaching medial PFC bilaterally, lateral premotor and PFC, insula, TPJ, IPL and SPL, and putamen sub cortically (Figure 3B). The associated white matter tracts included SLF III connecting TPJ/IPL to ventral frontal cortex; arcuate fasciculus connecting temporal to frontal cortex; fronto-insular tracts connecting at short distance the insula with the inferior frontal lobe; and frontal inferior longitudinal fasciculus within the frontal lobe. The cortical networks most affected were association networks such as the VAN, CON and DMN, but also auditory (Figures 4, 5).

Herein, we will consider the possible contribution to AHP of differently disconnected regions/tracts within and beyond the motor system.

The insula is considered crucial for monitoring of internal body functions: the anterior part for human awareness of all feeling from the body containing not only interoceptive but also a somatotopic representation of subjective feeling of moving (Craig, 2009); while the posterior part for sense of ownership, agency and body schema (Karnath, 2005; Tsakiris et al., 2007; Karnath and Baier, 2010). The disconnection of fronto-insular tracts may mediate the abnormal body signal perception and awareness in AHP patients.

The inferior frontal gyrus includes nodes belonging to different networks such as the CON, a control network that includes insula and cingulate cortex and that is involved in task maintenance and shifting (Sestieri et al., 2014). Therefore, one aspect of AHP may be the inability to shift out of incorrect signals possibly related to motor planning or body schema.

Although the ventral premotor area was disconnected in AHP, pre-SMA and SMA were spared. This is in line with the observation of Berti et al. (2005) who claimed that AHP reflects an inability to recognize sensory feedback notwithstanding a normal motor forward plan. However, damage to the CON and fronto-aslant tract does not rule a disconnection of pre-SMA and SMA.

The inferior frontal gyrus is also linked to the TPJ-supramarginal gyrus via SLFIII as part of the VAN. SLFIII was one of the most damaged white matter tracts. These regions/tracts may be relevant in AHP due to their role in integrating multi-modal body and visuospatial signals, and in switching between internal, bodily or self-perspective and external, or environmental, or others-perspective (Corbetta et al., 2008).

Regions in superior parietal and dorsal frontal cortex were also involved. These regions mediate top-down control signals for spatial attention (Corbetta et al., 2008). This damage may be related to the possible association of AHP with spatial neglect. Neglect symptoms were present indeed in a substantial part of our cohort of AHP patients (Table 2).

There is also disconnection of the angular gyrus and ventromedial PFC, bilaterally through the anterior corpus callosum. These are key nodes of the DMN, which might contribute to AHP through a number of internal processes including memory and self-referential behavior (Raichle et al., 2001) and emotional regulation (Raichle, 2015). Moreover, the bilateral involvement of ventromedial PFC could suggest that some aspects of anosognosia could not be related solely to right hemisphere dysfunction.

As previously described, for anosognosia a predominant role has always been recognized to the right hemisphere, nevertheless, this assumption may derive from its under-recognition in left hemisphere aphasic patients (Cocchini et al., 2009; Della Sala et al., 2009). Even though no left brain damaged AHP patient was present, we see evidence of bilateral prefrontal disconnection through the anterior corpus callosum.

Recently two studies have highlighted the role of white matter tracts and cortical regions around a wide fronto-temporo-parietal area. Moro et al. (2016) with a lesion voxels analysis on a large sample of AHP patients (*n* = 70) found acute AHP to be associated with damage of ventral premotor cortex, insula and superior temporal gyrus, basal ganglia and white matter tracts as superior corona radiata, arcuate fasciculus and ventral part of SFL. Ventral premotor and superior temporal damage along with associated white matter tracts were present in chronic cases of anosognosia. We found similarly a lesion pattern and structural disconnection for AHP pointing to cortical areas such as ventral premotor cortex, insula and temporal regions, and among white matter tracts also arcuate fasciculus and SLFIII.

Pacella et al. (2019) used a similar structural disconnection approach on a cohort of 95 patients with AHP. Lesions were directly measured and there were available clinical and neuropsychological information for co-variate analyses. There are also differences between our analysis and Pacella et al. (2019) in the dataset of healthy subjects (164 vs 10), and the threshold (0.5 vs no threshold).

They found disconnection of white matter tracts like SLF III, frontal-aslant pathways and cingulum, and at the network level disconnection of premotor/motor regions, limbic system, and VAN. Our analysis finds similar results: SLF III and FIL, AF, fronto-insular tracts, and fronto-aslant tract, with the most involved networks default mode (limbic), VAN, and cingulo-opercular that includes ventral premotor regions.

These studies jointly emphasize the neural correlates of AHP cannot be localized to a single brain region or functional system, e.g., motor-premotor. Rather, AHP as a syndrome involves several fronto-temporo-parietal areas and subcortical white matter, and multiple networks related to motor and body monitoring but also attention and self-referential processes. This in turn may connect with other theories on AHP as an aberrant predictive coding (Friston, 2005) due to a dynamic imbalance between prior beliefs, sensory feedbacks and prediction errors through large-scale networks (Fotopoulou, 2014).

One interesting question is whether this wide network disconnection is specific to AHP or partly generalizes to other forms of anosognosia. The current belief is that different forms of anosognosia are anatomically separate. It would be fascinating to find common anatomical areas or pathways with other forms, e.g., Anton or anosognosia for neglect.

Another interesting aspect that could be addressed in the future with functional studies of anosognosic patients are the temporal aspects of awareness. AHP is a fleeting syndrome that fluctuates over time. The factors controlling these fluctuations are unknown, but on-line monitoring of physiological activity through fMRI or EEG in parallel with behavioral assessments may show the dynamic loss of functional integration at moments of anosognosia.

## Conclusion

This study supports the hypothesis that AHP is a multicomponent network syndrome that includes multiple cognitive, as well as motor/premotor, networks.

## Data AVailability Statement

The datasets generated for this study are available on request to the corresponding author.

## Ethics Statement

Ethical review and approval was not required for the study on human participants in accordance with the local legislation and institutional requirements. Written informed consent for participation was not required for this study in accordance with the national legislation and the institutional requirements.

## Author Contributions

MC and EM planned the research. EM, FB, AB, and AS reviewed the literature and drew the lesions. AS carried out the lesion-based and disconnection-based comparison analysis. MB carried out analyses related to white matter tract-overlap and cortical parcellation. EM, FB, AS, MB, and MC wrote the manuscript. MC provided supervision across the whole study.

## Conflict of Interest

The authors declare that the research was conducted in the absence of any commercial or financial relationships that could be construed as a potential conflict of interest.

## Footnotes

1 http://www.fmrib.ox.ac.uk/analysis/techrep

2 www.mayoclinic.org

3 http://fsl.fmrib.ox.ac.uk/fsldownloads

4 http://www.itksnap.org/pmwiki/pmwiki.php?n=Downloads.SNAP3

5 http://fsl.fmrib.ox.ac.uk/fsldownloads

6 http://fsl.fmrib.ox.ac.uk/fsldownloads

7 http://www.fmrib.ox.ac.uk/fsl/

8 http://www.fmrib.ox.ac.uk/fsl/
