# Supplementary material for: Multiple Network Disconnection in Anosognosia for Hemiplegia
Source: Front Syst Neurosci. 2020 Apr 29;14:21. doi: 10.3389/fnsys.2020.00021 (PMC7201993; doi:10.3389/fnsys.2020.00021)
Supplement: Supplementary file 1 [file Data_Sheet_1.PDF]

Supplementary Material

Table 1 Supplementary- Demographical, clinical and radiological data of AHP patients: data of AHP patients (in case of group data the mean value is reported for every patient). Side of lesion: Right (R), Left (L). Age, in years. Sex: Male (M), female (F), not specified (ns). CT scan: present (1), absent (0), Reconstruction. MRI: present (1), absent (0). N° slices available. Volume (lesion in mm^3). Imaging after stroke (time in days). Type of stroke: Ischemic (I), Haemorrhagic (H). Awareness evaluation.

Article	Side of lesion	Age	Sex	CT scan	MRI	Reconstruction	n° slices available	Volume (mm^3)	Imaging- delay after stroke (days)	Type of stroke	Awareness evaluation
Piedimonte et al. 2018	R	80	ns	0	0	1 (MNI)	3	4352	ns	ns	Berti
Piedimonte et al. 2018	R	75	ns	0	0	1 (MNI)	4	768	ns	ns	Berti
Facchin et al. 2018	R	64	M	0	0	1 (MNI)	7	12744	90	I	VATA-m, AHP questionnaire
Piedimonte et al. 2015	R	66	M	0	0	1 (MNI)	10	28272	14	I	AHP questionnaire
Di Vita et al. 2015	R	63	M	0	1	0	3	33984	30	I	Bisiach
Besharati et al. 2014	R	88	F	1	0	0	8	52832	ns	I	Feinberg, Berti
Besharati et al. 2014	R	70	M	1	0	0	8	45272	ns	H	Bisiach, VATA-m, modified Marcel
Venneri et al. 2012	R	68	F	0	1	0	8	67160	60	I	Feinberg

Cogliano et al. 2012	R	59	M	1	0	0	2	5600	ns	H	Marcel
Fotopoulou et al. 2011	R	65	F	0	0	1 (MNI)	8	44144	ns	I	Ns
Fotopoulou et al. 2009	R	67	F	1	0	0	1	2840	1	ns	Berti Awareness, AHP questionnaire, Bi-manual, Bi-pedal,
Fotopoulou et al. 2008	R	63,75	ns	1	0	0	8	11648	20	ns	Berti, Feinberg, AHP awareness, Bisiach
Fotopoulou et al. 2008	R	63,75	ns	1	0	0	8	102672	20	ns	Berti, Feinberg, AHP awareness, Bisiach
Fotopoulou et al. 2008	R	63,75	ns	0	1	0	8	78232	20	ns	Berti, Feinberg, AHP awareness, Bisiach
Fotopoulou et al. 2008	R	63,75	ns	0	1	0	8	6544	20	ns	Berti, Feinberg, AHP awareness, Bisiach
Turnbull et al. 2005	R	68	M	0	0	1 (Damasio)	6	9952	ns	ns	House and Hodges
Venneri et al. 2004	R	85	F	0	1	0	4	9176	730	H	Feinberg, unimanual vs bimanual test
Morin et al. 2003	R	51	M	0	1	0	1	11032	300	I	Bisiach

Berti et al. 2000	R	77	F	0	0	1 (Damasio)	9	84768	30	I	ns
Tei H. 2000	R	76	F	1	0	0	4	2704	ns	I	ns
Gil et al. 1995	R	93	F	1	0	0	4	13528	ns	ns	ns
House et al. 1988	R	89	F	1	0	0	2	2624	6	I	ns
Cappa et al. 1987	R	57	M	1	0	0	4	736	1	H	Bisiach, test for personal neglect
Salvato et al. 2016	R	89	F	0	0	1 (MNI)	8	53960	23	I	Nimmo-Smith, Marcel, & Tegnér
Van Stralen et al. 2011	R	60	F	1	0	0	3	13872	ns	H	Questionnaire for deficit in body ownership
Moro et al 2014	R	65	M	1	0	0	7	22864	ns	H	Bisiach
Moro et al 2014	R	63	M	1	0	0	7	2320	ns	H	Bisiach
Moro et al 2014	R	63	M	1	0	0	7	20504	ns	I	Bisiach

Table 2 Supplementary. Demographical, clinical and radiological data of AHP and HP patients (n=19).

(NS= not specified, I= Ischemic, H= Haemorrhagic, I+H= Ischemic with haemorrhagic infarction)

Demographic, clinical and radiological data	AHP Patients	HP Patients
Selected Subjects	28	19
Side of lesion R/L	28/0	19/0
Mean age (Yr.)	69,85 ± 12	55,5 ± 10,0
Sex F/M	11/11, 6 NS	12/7
CT scan	14	NS
MRI	6	19
Reconstruction (MNI/Damasio)	8	0
Mean imaging- delay after stroke (D)	91,0 ± 191,7	15,2 ± 6,6
Lesion Volume (mm^3)	26610,8±28693,1	87603,7 ± 86154,9
Type of stroke I/H	12/7, 9 NS	14/3, 2 I+H

Table 3 Supplementary - Clinical features of AHP and HP patients (n=19).

(0 if absent or not specified)

Clinical feature	AHP Patients	HP Patients
Selected Subjects	28	19
Sensory Impairment	14	12
Visual Impairment	12	7
Neglect	22	11
Somatoparaphrenia	7	0
Asomatognosia	7	0
Anton syndrome/ Anosognosia for Visual Impairment	2	0
Anosognosia for Hemianesthesia	3	0
Others*	6	0

*e.g.: anosognosia for neglect, allochiria, anosodiaphoria, hyperkinetic motor behaviour, halien hand, misoplegia

Figure 1 Supplementary. Mapping of disconnection maps on subcortical areas. HP vs AHP: subcortical areas overlap with disconnectome map, (subcortical area/disco in blue).

Figure 2 Supplementary. Lesions frequency map: Voxel lesion overlap in AHP (orange-yellow) and HP groups (n=19)(blue-teal). The color scale indicates the max number of patients with lesions in one voxel.

Figure 3 Supplementary. **Structural disconnection frequency map (75%):** Voxels of white matter tract overlap in AHP (orange-yellow) and HP (n=19) (blue-teal) groups. The color scale indicates max number of patients per voxel.

Figure 4 Supplementary. Lesion-based mapping: voxels with significant difference in lesion frequency between HP (n=19) and AHP patients. HP>AHP ($p<0.05$) (blue-teal). No voxels for AHP>HP damage.

Figure 5 Supplementary. Disconnection-based maps: voxels showing significant difference in white matter disconnection. HP>AHP (blue-teal). AHP>HP (orange-yellow). From top to bottom ($p<0.05$, $p<0.10$, $p<0.20$).
