# Supplementary figures and images for: Multiple Network Disconnection in Anosognosia for Hemiplegia
Source: Front Syst Neurosci. 2020 Apr 29;14:21. doi: 10.3389/fnsys.2020.00021 (PMC7201993; doi:10.3389/fnsys.2020.00021)

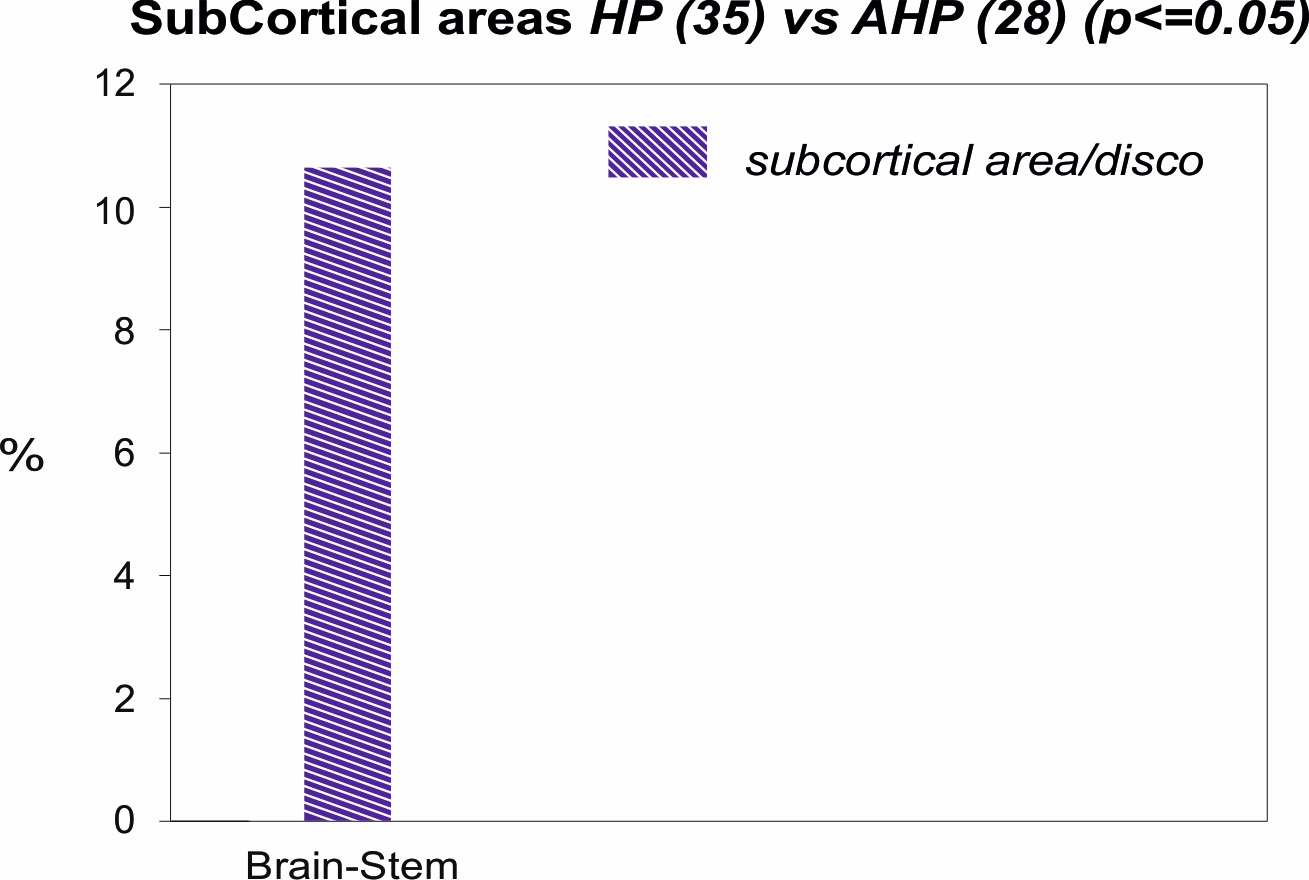

Supplement: Supplementary file 2 [file Image_1.jpeg]

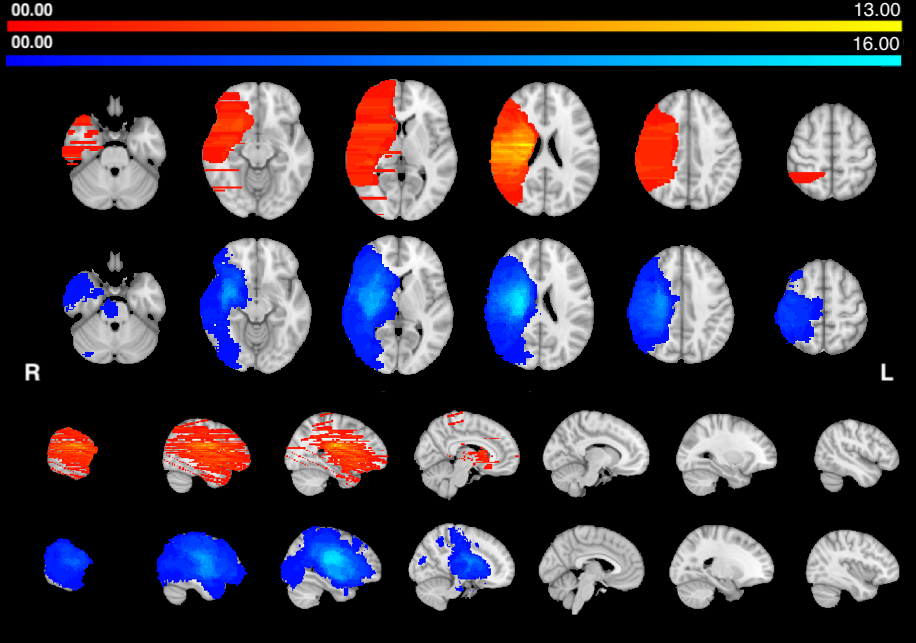

Supplement: Supplementary file 3 [file Image_2.jpeg]

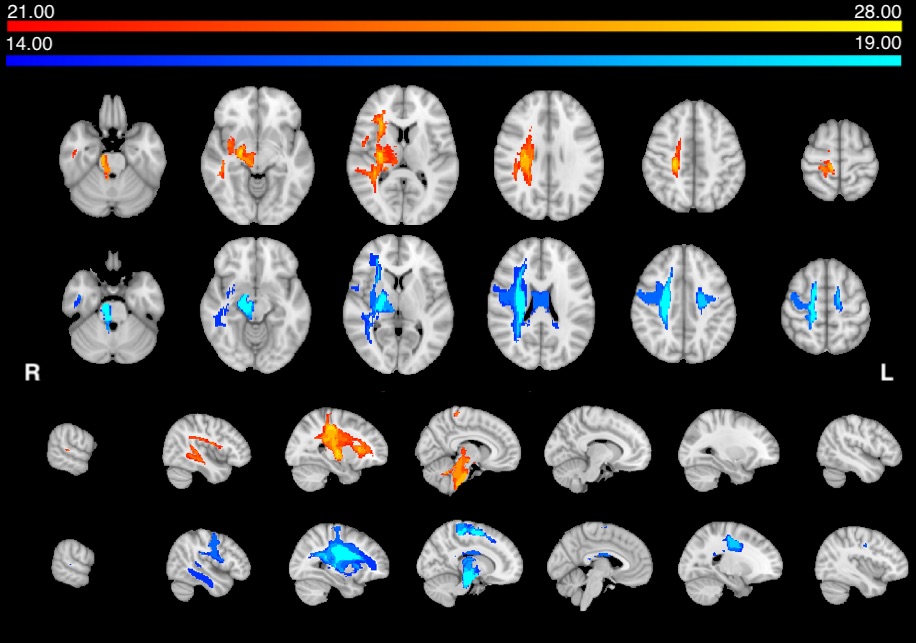

Supplement: Supplementary file 4 [file Image_3.jpeg]

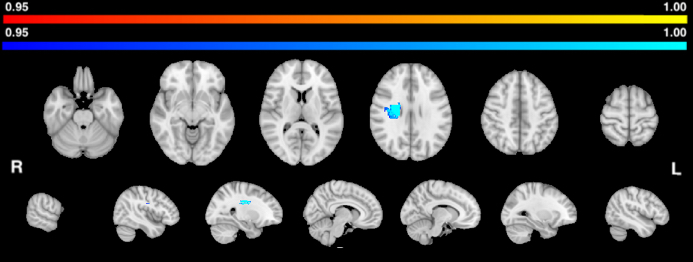

Supplement: Supplementary file 5 [file Image_4.jpeg]

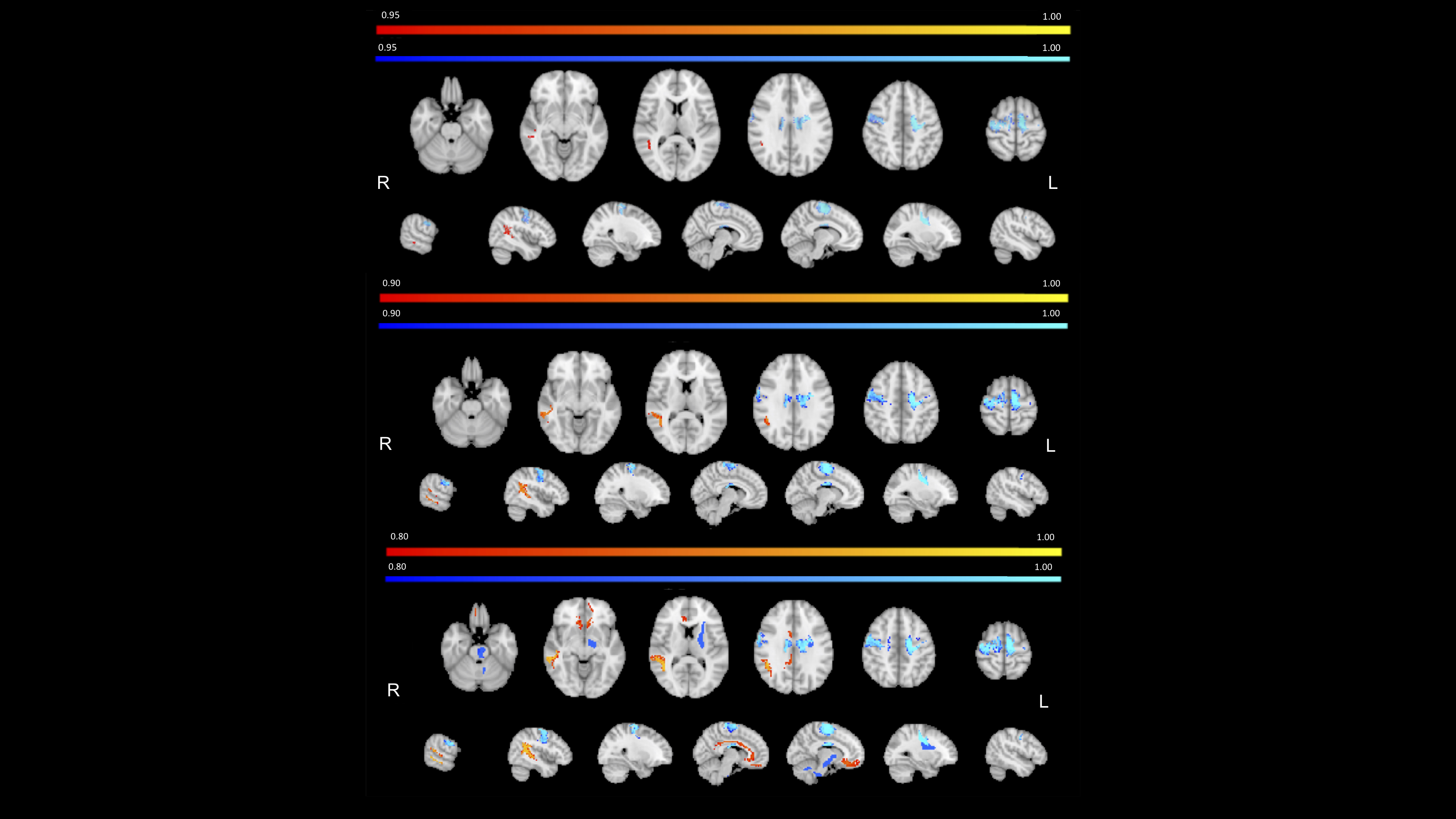

Supplement: Supplementary file 6 [file Image_5.jpg]
